# Understanding persistent GP turnover using work and personal characteristics: a retrospective observational study

**DOI:** 10.3399/BJGP.2025.0260

**Published:** 2026-01-27

**Authors:** Laura Jefferson, Ben Walker, Rosa Parisi, Matt Sutton, Evangelos Kontopantelis, Katherine Checkland

**Affiliations:** 1 Health Organisation, Policy and Economics (HOPE) Group, Centre for Primary Care and Health Services Research, University of Manchester, Manchester, UK; 2 Division of Population Health, Health Services Research and Primary Care, School of Health Sciences, University of Manchester, Manchester, UK; 3 Division of Informatics, Imaging and Data Sciences, School of Health Sciences, University of Manchester, Manchester, UK

**Keywords:** general practice, primary health care, job satisfaction, personal satisfaction, workforce

## Abstract

**Background:**

Rising GP turnover, declining participation rates, and growing workforce pressures threaten the sustainability of general practice. As policy shifts towards community-based care and workforce retention, understanding the job characteristics linked to high turnover is crucial.

**Aim:**

To examine the relationship between practice-level persistent GP turnover and GP job satisfaction.

**Design and setting:**

A retrospective observational study was conducted using linked national administrative data and survey responses for GPs in England.

**Method:**

Annual national GP workforce datasets were linked for 2013–2019 to calculate GP turnover, defining persistent high-turnover practices as those where over 10% of GPs left each year in three consecutive years. This information was merged with responses of individual GPs participating in the national GP Worklife Survey (GPWLS) in 2015, 2017, and 2019. Multiple linear regression analyses were used to relate work satisfaction components (including composite scores ‘autonomy’, ‘belonging’, and ‘competence’) to persistent turnover.

**Results:**

Among 2403 GPs, 8% worked in persistent high-turnover practices. After adjusting for covariates, these GPs reported significantly lower sense of autonomy, belonging, and competence in their roles, and lower overall job satisfaction, life satisfaction, and higher working hours. Notably lower scores were found for elements of the role related to GPs’ sense of competence.

**Conclusion:**

A clear relationship exists between GP job satisfaction and high turnover. The notable differences in experiences in some key work characteristics suggest targets for developing interventions supporting GP retention.

## How this fits in

GP turnover rates from national administrative datasets have previously been used to explore practice-level factors associated with turnover and its relationship to patient outcomes. The individual and work characteristics associated with turnover is less well understood, with much research focusing on intentions to leave or smaller samples of GPs leaving practice. This study sought to fill this research gap, through analysis of a large dataset of GPs’ working experiences linked to turnover, understanding potential predictors that may offer solutions to the workforce crisis being faced in general practice. This study found that GPs’ sense of autonomy, belonging, and competence are significantly lower in practices with problems with persistent turnover. It has also demonstrated how satisfaction with work characteristics, such as working hours and experiences of strained relationships, differs in practices with persistent turnover.

## Introduction

An imbalance exists in general practice in terms of healthcare workforce capacity, rising patient demand,^1–4^ and long waiting lists for secondary care^5^ in the wake of the COVID-19 pandemic, which has led to unsustainable pressure on the general practice workforce. As the ‘front door’ to the NHS, the workforce crisis facing general practice has profound implications for the sustainability of the health system and patient care.

Capacity is impacted by the inflow of healthcare professionals (through UK-based training or international recruitment), participation rates, productivity, and workforce attrition. Despite previous government efforts to increase GP numbers through pledges to recruit 6000 more GPs^6^ and ambitious recruitment plans outlined in the NHS Long Term Workforce Plan,^7^ training more doctors takes time. A critical shift in policy focus has therefore been made towards retention of the existing workforce. The Royal College of General Practitioners (RCGP) has stressed a need for widespread implementation of retention schemes as a key priority to support existing GPs,^8^ while Lord Darzi’s 2024 report called for a rebalance in funding flows with greater investment in community approaches, highlighting a downward trajectory in relative share of NHS expenditure on primary care from 24% in 2009 to 18% in 2021.^9^

Turnover rates among GPs were rising in the 10 years before the pandemic,^10^ exacerbated subsequently by GP burnout,^11,12^ mounting workload pressures and leading to increased intentions to leave practice.^13^ A recent RCGP ballot has suggested 40% of GPs are considering leaving the profession,^14^ while other estimates have suggested one-third of GPs may leave direct patient care within 5 years, with notable recent increases in the under 50-years age group.^13^ Workforce data have indicated that GPs are choosing to reduce their working hours or retire early, leading to declining contracted hours; for example, from 2015 to 2022 median full-time equivalents reduced from 0.80 to 0.69.^4^ One analysis has projected shortfalls of up to one in four GP posts by the end of the decade.^15^ This has far-reaching implications for the stability of the healthcare system, patient continuity, and quality of care.

In England, national administrative data are collected about the primary care workforce, which is a valuable resource that has previously been used to demonstrate variation in GP turnover rates over time and practice-level factors associated with turnover.^10^ There is a lack of evidence, however, on the individual-level factors in GPs’ working lives and personal characteristics that may help build our understanding of key factors associated with turnover. In a system with varying practice organisations, local population needs, and GP working preferences, there can be no ‘one-size-fits-all’ approach, so understanding these individual-level predictors of retention is needed.

## Method

Using linked national administrative data on persistent turnover of GPs in English general practice and large GP cohort datasets from the GP Worklife Surveys (GPWLS),^13,16–19^ associations were examined between high practice turnover and GP job satisfaction.

### Data

#### Persistent GP turnover

GP turnover was calculated using linked GP workforce datasets, including NHS Digital (now NHS England) and GPs by general practice (GP membership-epracmem, available from the NHS Prescription Data Service [NHS RxS] and published by the NHS Organisation Data Service [ODS]). These contained practice-level characteristics and dates that individual GPs joined or left a practice. Practices were defined as having ‘persistent high GP turnover’ if more than 10% of GPs left each year for three consecutive years.^10^ Data from 2013–2015, 2015–2017, and 2017–2019 were used to correspond with survey datapoints. More details on the data sources used are in Supplementary Table S1.

#### GP characteristics and experiences

GP characteristics and experiences (including job satisfaction and job pressures) were obtained from the GPWLS. The GPWLS is a biennial survey of GPs in England that has been running since 1998. The survey asks participants about their working lives, including work-related satisfaction and stress, as well as demographic and practice characteristics.

Until 2019, there was a cross-sectional element (involving a random sample of all GPs included in the national administrative data [‘Current GP practitioners in England and Wales’: egpcur.csv])^20^ and a longitudinal element (inviting all responders to the previous survey to participate again). To ensure comparability over time, responses were used from all responders to the cross-sectional elements of the 2015, 2017, and 2019 surveys in the present analysis. Using the practice ID code, GPs working at persistently high-turnover practices were identified to enable comparisons.^16–19^

#### Outcomes

Components of the GPWLS selected for analyses were pre-defined by topic experts (first, fourth, and sixth authors) on the basis of previous research and theoretically informed by the ‘ABC of doctors’ needs’ framework developed by West and Coia,^21^ based on Deci and Ryan’s self-determination theory.^22^ This framework advocates the need for autonomy, belonging, and competence to support doctors’ wellbeing at work, which maps to several components of the GPWLS from the job satisfaction, job pressures, and job characteristics sections. Table 1 indicates the selected items and how these were grouped to create three composite outcome variables, using average scores for relevant items.

**Table 1.** GP Worklife Survey items included in ABC composite scales and scaling of items

Component	Original scale	Reversed (Yes or no)
**Autonomy components**		
Satisfaction with physical conditions	1–7 Likert scale: 1 = extremely dissatisfied, 7 = extremely satisfied	No
Satisfaction with freedom to choose method of working	1–7 Likert scale: 1 = extremely dissatisfied, 7 = extremely satisfied	No
Satisfaction with responsibility	1–7 Likert scale: 1 = extremely dissatisfied, 7 = extremely satisfied	No
Satisfaction with opportunities	1–7 Likert scale: 1 = extremely dissatisfied, 7 = extremely satisfied	No
Satisfaction with hours of work	1–7 Likert scale: 1 = extremely dissatisfied, 7 = extremely satisfied	No
Satisfaction with amount of variety in your job	1–7 Likert scale: 1 = extremely dissatisfied, 7 = extremely satisfied	No
Pressure at work: paperwork	1–5 Likert scale: 1 = no pressure, 5 = high pressure	Yes
Consulted about changes that affect my work	1–5 Likert scale: 1 = strongly disagree, 5 = strongly agree	No
**Belonging components**		
Satisfaction with colleagues	1–7 Likert scale: 1 = extremely dissatisfied, 7 = extremely satisfied	No
Satisfaction with recognition	1–7 Likert scale: 1 = extremely dissatisfied, 7 = extremely satisfied	No
Satisfaction with remuneration	1–7 Likert scale: 1 = extremely dissatisfied, 7 = extremely satisfied	No
Relationships at work are strained	1–5 Likert scale: 1 = strongly disagree, 5 = strongly agree	Yes
**Competence components**		
Increased demand from patients	1–5 Likert scale: 1 = no pressure, 5 = high pressure	Yes
Dealing with problem patients	1–5 Likert scale: 1 = no pressure, 5 = high pressure	Yes
Worrying about complaints or litigation	1–5 Likert scale: 1 = no pressure, 5 = high pressure	Yes
Insufficient time to do justice to job	1–5 Likert scale: 1 = no pressure, 5 = high pressure	Yes
Long working hours	1–5 Likert scale: 1 = no pressure, 5 = high pressure	Yes
Increasing workloads	1–5 Likert scale: 1 = no pressure, 5 = high pressure	Yes
**Other scores**		
Overall job satisfaction	1–7 Likert scale: 1 = extremely dissatisfied, 7 = extremely satisfied	No
Overall life satisfaction	1–7 Likert scale: 1 = extremely dissatisfied, 7 = extremely satisfied	No

Autonomy relates to the need for doctors to experience control in their professional roles,^23^ as such items relating to the following were included: satisfaction with physical conditions; freedom to choose methods of working; opportunities to use abilities; hours of work; variety in work; paperwork; and consultation about changes in work. Belonging relates to doctors’ need to feel connected to others in the workplace and feel valued in their working lives,^23^ with GPWLS items ascertaining to relationships with colleagues and recognition being included in this composite variable. Competence relates to doctors’ perceived ability to perform effectively and sense of delivering valued outcomes,^23^ as such items were included relating to patient demand, managing patients and complaints, insufficient time, and workload. Cronbach’s alpha for internal consistency of components of the composite scores were 0.83 (autonomy), 0.72 (belonging), and 0.78 (competence).

Self-reported overall job satisfaction, self-reported life satisfaction, and hours worked outcome variables were also included. For ease of interpretation, each component was rescaled and, where necessary, they were reversed, to create a score from 0–100, where 0 = worst possible outcome (for example, lowest level of autonomy) and 100 = best possible outcome (for example, best level of autonomy).

#### Statistical analyses

Linear regression was used to relate working in a persistently high-turnover practice to the six outcomes, adjusting for GP characteristics and survey year. For exposure, the study used whether a GP was working in a practice classified as persistently high turnover at the time of response to the survey. A series of individual-level covariates were adjusted for, which have been used in previous studies exploring GP workforce satisfaction, including self-identified gender, marital status, ethnicity (categorised as White or all other ethnic groups combined owing to small samples for some ethnic backgrounds), whether they had a child aged ≥18 years, contract status (salaried versus partner, other categories excluded owing to small sample sizes), and age.^19,23–25^ Standard errors were clustered at practice level. Given the exploratory nature of this analysis, the study examined both composite scores and responses to individual items to better understand variation across specific components within each domain.

As work hours could feasibly influence wellbeing, a sensitivity analysis was conducted where the models were re-estimated that had autonomy, belonging, competence, overall job satisfaction, and overall life satisfaction as outcomes, and adjusted for hours worked per week. Owing to satisfaction with hours of work being a component of the autonomy score, and pressure from long working hours being a component of the competence score, these are not presented as main results.

#### Missing values

Multiple imputation by chained equations were conducted to address missingness in the outcomes and covariates.^26^ The imputation model closely aligned to the analytical model, with all outcome components, the exposure, and all covariates used for the imputation. In Supplementary Table S2 missingness for each variable before imputation are reported.

## Results

### Descriptive statistics

The sample included 2403 GPs, 8.0% of whom worked in persistently high-turnover practices (*n* = 193). The characteristics of GPs who worked in persistently high-turnover practices are compared with those who did not in Table 2. The sample included almost equal proportions of men and women (49.4% men), most of the sample had a child aged ≥18 years (59.4%), and the mean age was 48.2 years (standard deviation [SD] 9.02). The majority were from a White ethnic background (78.3%). Most responders were GP partners (81.5%). In persistently high-turnover practices there was a far higher percentage of women (61.7%) relative to those who worked at other practices (49.6%). GPs in persistent high-turnover practices were also on average slightly younger and worked in practices with fewer partners.

**Table 2.** Descriptive statistics

	Other practices, ***n*** **= 2210**		Persistently high-turnover practices, ***n*** **= 193**		Total, ***N*** **= 2403**	
**Variable**	**Mean or** ***n***	**SD or %**	**Mean or** ***n***	**SD or %**	**Mean or** ***n***	**SD or %**
**Autonomy**						
Satisfaction with physical working conditions	70.1	23.9	64.9	27.1	69.6	24.2
Satisfaction with freedom to choose own method of working	61.6	24.2	56.1	25.9	61.2	24.4
Satisfaction with responsibility	64.9	27.1	57.6	28.1	64.3	27.2
Satisfaction with opportunity to use abilities	66.1	23.7	63.6	23.9	65.9	23.7
Satisfaction with hours of work	44.5	29.8	36.8	29.3	43.9	29.8
Satisfaction with variety in job	69.5	22.6	67.8	24.3	69.4	22.7
Satisfaction with paperwork	17.4	21.9	12.9	20.6	17.0	21.8
Choice in how to perform job	56.8	25.2	53.6	26.5	56.5	25.3
Choice in deciding what I do at work	48.6	25.2	44.5	26.1	48.3	25.3
Consulted about changes	49.6	30.8	45.8	31.2	49.3	30.8
Autonomy score	62.8	18.1	57.8	19.0	62.4	18.2
Autonomy score sensitivity	54.9	16.0	50.4	17.0	54.5	16.1
**Belonging**						
Satisfaction with colleagues	78.5	20.5	76.1	20.8	78.3	20.5
Satisfaction with recognition for good work	56.9	26.2	50.0	25.8	56.3	26.2
Satisfaction with remuneration	55.2	27.3	49.7	27.1	54.7	27.3
Strained relationships	62.7	27.9	55.2	27.0	62.1	27.9
Belonging score	63.5	19.4	58.6	18.9	63.1	19.4
Belonging score sensitivity	63.3	18.8	57.7	18.1	62.9	18.8
**Competence**						
Pressure from patient demands	18.2	19.1	15.2	18.5	18.0	19.0
Pressure from problem patients	27.2	23.4	24.6	23.0	27.0	23.4
Pressure from worry about complaints	35.8	28.1	29.5	28.0	35.3	28.1
Pressure from insufficient time	16.5	21.7	10.3	16.5	16.0	21.4
Long working hours	24.2	26.9	18.6	24.1	23.7	26.7
Competence average	24.4	17.5	19.7	15.6	24.0	17.4
**Other scores**						
Job satisfaction	55.1	24.8	48.9	25.9	54.6	25.0
Life satisfaction	64.0	24.0	58.9	26.3	63.6	24.2
Hours worked	40.6	14.2	42.6	14.3	40.8	14.2
**Covariates**						
Gender						
Men	1113	50.4%	74	38.3%	1187	49.4%
Women	1097	49.6%	119	61.7%	1216	50.6%
Has child aged ≥18 years						
No	900	40.7%	76	39.4%	976	40.6%
Yes	1310	59.3%	117	60.6%	1427	59.4%
Age	48	9.05	47.01	8.64	48.23	9.02
Marital status						
Not married or cohabiting	219	9.9%	22	11.4%	241	10.0%
Married or cohabiting	1991	90.1%	171	88.6%	2162	90.0%
Ethnicity						
Other than White	486	22.0%	35	18.1%	521	21.7%
White	1724	78.0%	158	81.9%	1882	78.3%
Year of survey						
2015	913	41.3%	90	46.6%	1003	41.7%
2017	801	36.2%	68	35.2%	869	36.2%
2019	496	22.4%	35	18.1%	531	22.1%
Partner						
No	402	18.2%	42	21.8%	444	18.5%
Yes	1808	81.8%	151	78.2%	1959	81.5%

SD = standard deviation.

**Figure 1.**
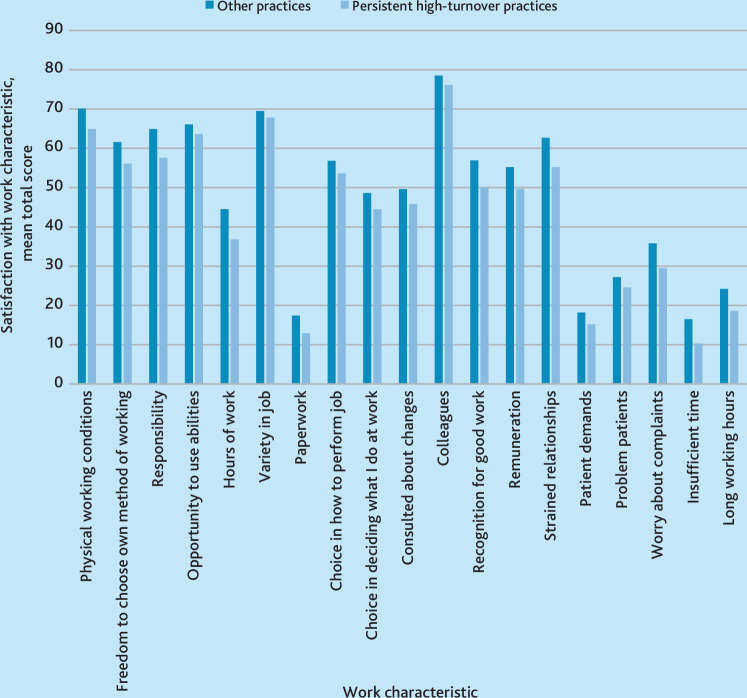
Variation in satisfaction with work characteristics by practice turnover

Figure 1 demonstrates the variation in satisfaction with different work characteristics and compares GPs in persistent high-turnover practices with those in other practices. Across all work characteristics, the lowest scoring was satisfaction with paperwork and the highest scoring was relationships with colleagues, for both persistent high-turnover practices and other practices.

For the components of the ‘autonomy’ domain, the item with the lowest average total score was paperwork, which was noticeably lower among GPs at persistently high-turnover practices. Satisfaction with physical working conditions and variety in the job had the highest mean score in this domain, although satisfaction with physical working conditions was lower in GPs at persistent high-turnover practices than other practices. GPs’ satisfaction with freedom to choose their own method of working and responsibility were also notably lower among those working in practices with persistent high turnover (Figure 1).

In terms of work characteristics in the ‘belonging’ domain, GPs scored lowest in their satisfaction with remuneration, while relationships with colleagues scored the highest in total. There were notably lower mean scores for several items in the belonging domain for GPs in persistent high-turnover practices, including recognition for good work and strained relationships (Figure 1 and Table 2).

For the ‘competence’ domain, the lowest scoring items on average were those relating to workload, including having insufficient time and patient demands, while the highest average score in this domain related to concerns about patient complaints (with higher scores indicating lower concerns) (Figure 1).

### Characteristics associated with persistent high turnover

**Figure 2.**
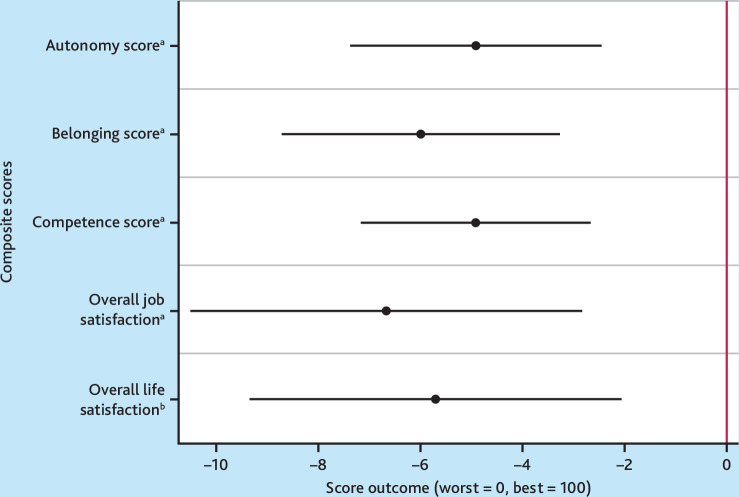
Regression results, *N* = 2403. Bars represent 95% confidence intervals. Figure shows results from ordinary least squares (OLS) regressions, where 0 indicates no difference between scores and the binary exposure variable, working at a high-turnover practice. ^a^ *P*<0.001*.* ^b^ *P*<0.01.

The study found that after adjusting for covariates, working in a persistently high-turnover practice was significantly associated with a 4.9 lower autonomy score (*P*<0.001, 95% confidence interval [CI] = 2.4 to 7.4, cohen’s *d*: 0.285), 6.0 lower belonging score (*P*<0.001, 95% CI = 3.3 to 8.7, cohen’s *d*: 0.323), and 5.0 lower competence score (*P*<0.001, 95% CI = 2.6 to 7.2, cohen’s *d*: 0.316) (Figure 2).

In terms of job and life satisfaction, it was found that working in a persistently high-turnover practice was significantly associated with 6.7 lower overall job satisfaction (*P*<0.001, 95% CI = 2.8 to 10.5, cohen’s *d*: 0.252) and 5.7 lower life satisfaction (*P*<0.01, 95% CI = 2.0 to 9.4, cohen’s *d*: 0.224) (Figure 2). Working in a persistently high-turnover practice was also significantly associated with working 3.00 hours more per week (*P* = 0.003, 95% CI = 1.0 to 5.0, cohen’s *d*: 0.218), after controlling for other covariates (see Supplementary Figure S1).

The sensitivity analyses found that the relationship between working in a persistently high-turnover practice and autonomy, belonging, competence, and job and life satisfaction are robust to adjusting for hours worked per week, although slightly attenuated (see Supplementary Figure S1).

## Discussion

### Summary

The present study findings reveal that working in a persistently high-turnover practice is associated with lower overall job satisfaction and lower life satisfaction. The composite scores of characteristics associated with autonomy, belonging, and competence in GPs’ working lives reveal that GPs in persistent high-turnover practices report lower scores across these domains. Using standard guidelines for interpretation of effect size,^27^ the values indicate small to moderate effect sizes, suggesting that working in persistently high-turnover practices is associated with modestly worse scores across all domains. While the ‘ABC of doctors’ needs’ framework advocates these three core needs for a motivated, healthy workforce,^21^ the present findings also indicate these components may be eroded in practices experiencing persistent high turnover.

In terms of ‘autonomy’ — doctors’ need to have control over their work — lower GP satisfaction was particularly noticeable in relation to GPs’ hours of work and paperwork, although across all autonomy items GPs’ satisfaction was lower in practices with persistent turnover. Similarly, lower GP satisfaction scores were found in work characteristics comprising the ‘belonging’ score, which was related to doctors’ feelings of connectedness, being cared for, and valued. Work characteristics comprising the ‘competence’ composite variable — relating to doctors’ need to deliver effective health care and valued outcomes — scored among the lowest across all work characteristics. GPs reported greater pressures owing to patient demands, problem patients, worrying about complaints, and insufficient time across the board, but with lower scores in practices with persistently high GP turnover. GPs in a persistently high-turnover practice also worked 3 hours more per week.

### Strengths and limitations

A strength of this study is the use of detailed survey data from 2403 GPs, linking GP work and personal characteristics with practice-level information and turnover. Furthermore, the richness of the dataset allowed the authors to adjust for multiple individual-level characteristics. Multiple imputation was used to reduce the risk of bias through missing data.

Nevertheless, while this analysis tests perhaps intuitive assumptions about job satisfaction and GP turnover, the data do not allow causal inferences to be made; GP and role characteristics were co-occurring at practices with persistent high turnover, but it is not known if lower satisfaction scores were a consequence of high turnover or if these factors lead to more GPs leaving a practice. For example, higher proportions of women were found in practices with persistent turnover, but it cannot be inferred whether more women in a practice leads to higher turnover, or whether women become ‘stuck’ in persistent turnover practices. Previous research demonstrating women’s lower geographical mobility and the influence on women GPs’ career decisions suggests it may be the latter.^23^ Further research is needed. The present datasets did not contain information on why GPs left a practice, so it could not be determined whether they moved to another practice, left the profession, or retired.

While other demographic characteristics and GP role characteristics did not appear to be related to turnover, the present sample was limited in terms of ethnic diversity and underrepresented GPs from salaried and locum roles. Further research is needed to explore the experiences of a wider, more representative group. Owing to the repeated cross-sectional nature of the data, the study was restricted to what had been captured in previous surveys; there may be other individual-level characteristics that the study was not able to adjust for.

While individual item scores were presented to illustrate variation across components of each domain, it is acknowledged that this does not constitute formal validation of the composite measures. Future work should examine the dimensionality and construct validity of these composites more systematically.

Furthermore, while sensitivity analysis demonstrates the present findings are robust to adjusting for hours worked per week, the authors caution against interpreting these results as they likely underestimate the relationship owing to satisfaction with hours of work being a component of the autonomy score, and pressure felt from long working hours being a component of the competence score.

Finally, GPWLS survey data were used up to 2019, capturing sentiment and experiences before the COVID-19 pandemic. During this time the GP workforce experienced significant challenges,^11^ intentions to leave increased,^13^ and GPs reduced their working hours.^4^ At the time of analysis, GPWLS 2021 data were available but excluded since this was a COVID-19 year; therefore, further analyses are needed to explore GP experiences and turnover using more recent datasets.

### Comparison with existing literature

Previous research has linked persistent high GP turnover with area level deprivation, larger practices, greater burden of serious health conditions, urban locations, and lower patient access.^10,28^ While the present analysis did not adjust for these characteristics, the study provides further information on GP and role characteristics related to turnover, giving a more complete picture about GPs’ experiences in practices with persistent turnover. Similar findings have been reported in studies of GP leavers’ experiences, with increased paperwork, bureaucracy and workload, lack of time, and diminished autonomy and professional identity associated with early career exit.^29,30^ Challenges of strained relationships in general practice teams resulting from implementation of remote and technological work have been recently reported,^31^ while Fisher and McDermott^32^ have stressed a need to improve practice cultures as a tool to support workforce retention. The present findings strengthen this call, highlighting noticeable differences in strained relationships and lowered sense of belonging in practice teams with persistent turnover.

### Implications for research and practice

As the ‘front door’ to the NHS, GP workforce retention has profound implications for the sustainability of the health system and patient care. The present findings have demonstrated a clear link between GPs’ sense of autonomy, belonging, and competence in their roles and workforce retention. Future research using longitudinal modelling and factor analysis may enable further insights, particularly since the ‘ABC of doctors’ needs’ framework, although based on Deci and Ryan’s self-determination theory,^22^ has not yet been validated empirically in this setting.

This analysis of a large sample of GPs has provided further emphasis for practices, networks, and government on the need to act to implement changes to support staff in these key domains. With significant financial costs estimated at >£295 000 to replace a GP post and £500 000 to train new GPs,^33,34^ research should also now support this action, providing evidence-based solutions. Existing interventions tested in research literature focus on recruitment^35^ or individual-focused behavioural techniques that situate the problem with the individual, rather than addressing broader organisational or system factors.^36^ Future intervention development should consider the key factors identified here as impacting GPs’ sense of autonomy, belonging, and competence.

## Data

The data are not publicly available due to restrictions as per research participant consent.
